# Response to ‘Monotreme middle ear is not primitive for Mammalia’

**DOI:** 10.1093/nsr/nwab132

**Published:** 2021-07-23

**Authors:** John R Wible, Sarah L Shelley, Shundong Bi

**Affiliations:** Section of Mammals, Carnegie Museum of Natural History, USA; Centre for Vertebrate Evolutionary Biology, Yunnan University, China; Section of Mammals, Carnegie Museum of Natural History, USA; School of Geosciences, University of Edinburgh, UK; Section of Mammals, Carnegie Museum of Natural History, USA; Centre for Vertebrate Evolutionary Biology, Yunnan University, China; Department of Biology, Indiana University of Pennsylvania, USA

To date, a complete auditory apparatus (with malleus, incus, stapes, ectotympanic and perhaps surangular) is known for only three Mesozoic mammals, the haramiyidan *Arboroharamiya allinhopsoni* [1], the multituberculate *Sinobaatar pani* [2] and the zhangheotheriid *Origolestes lii* [3], with subsets of these bones preserved in a handful more, including the haramiyidan *Vilevolodon diplomylos* [4,5] discussed here. A goal of Wang *et al.* [5] was to evaluate the debate surrounding the various interpretations of these bones and to offer a cautionary tale about overinterpreting these structures. Because the new specimen of *Vilevolodon* reported by Wang *et al.* [5] preserved left and right mallei and incudes in articulation, these authors focused on the incudomallear joint. Wang *et al.* [5] found these two bones in *Vilevolodon* to be reminiscent of those in extant monotremes, with a relatively flat articulation between a plate-like incus and similarly thin malleus. On the strict consensus tree from their parsimony analysis, Wang *et al.* [5] optimized five characters of the incudomallear articulation and reported that the character states associated with the overlapping incudomallear articulation of extant monotremes and *Vilevolodon* optimized as primitive for Mammalia. However, they expressed uncertainty as to whether this overlapping joint evolved convergently in haramiyidans and monotremes or was an innovation at the level of Mammalia.

Meng and Mao [6] question Wang *et al.*’s [5] identification of the incus in the new specimen of *Vilevolodon*, because it differs from that reported for the holotype [4]. Wang *et al.* [5] addressed this already, noting that the two incudes have the same shape, with the only difference being the interpretation of the incudomallear joint. Meng and Mao [6] then conduct an optimization employing different criteria (their Fig. 1o) from those of Wang *et al.* [5]. Rather than optimizing the five characters individually, as had Wang *et al.* [5], Meng and Mao [6] lump the five into two broad morphologies: overlapping and partial overlapping joints (their braced hinge joint). They report that it is more parsimonious to have the partial overlapping joint as primitive for Mammalia, which they state falsifies Wang *et al.*’s hypothesis. Meng and Mao [6] illustrate what they consider to be the Wang *et al.* [5] hypothesis in their Fig. 1n, but we emphasize that this was not an analysis that was ever conducted by the latter authors.

**Figure 1.**
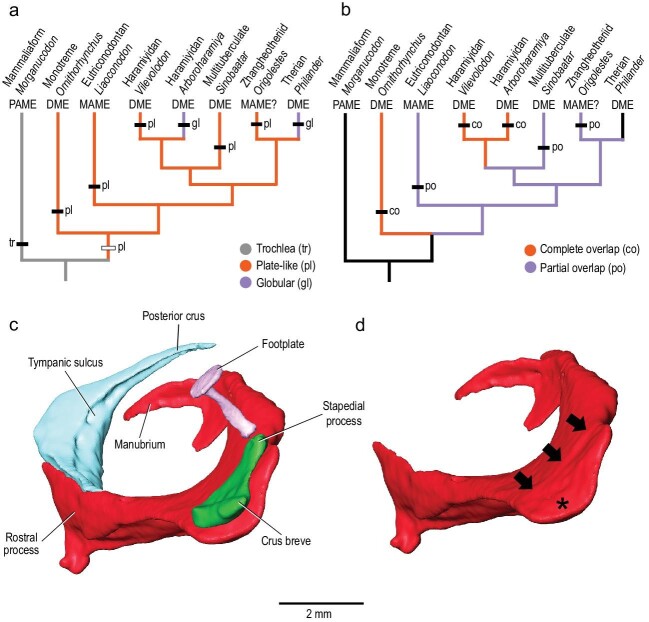
(a) Optimization of character 417 from Wang *et al.* [5], the shape of the incudal body, on simplified consensus tree, with plate-like identified as primitive for Mammalia. (b) Optimization of character 419 from Wang *et al.* [5], extent of overlap of malleus and incus (only applicable for taxa with the incudomallear articulation in the same plane as the mallear body—character 418), on simplified consensus tree, with the primitive condition ambiguous for Mammalia (see Supplementary Data online for terminology and abbreviation). (c and d) Isosurfaces from CT scan of the extant monotreme *Ornithorhynchus anatinus*, Carnegie Museum 50815: (c) malleus (red), incus (green), stapes (purple) and ectotympanic (light blue) in oblique posterodorsal view; (d) malleus (red) with black arrows on low ridge marking the border of the incudal facet and asterisk in the concave part of the incudal facet.

Wang *et al.* [5] did not figure any of their five individual optimizations. We revisit them here, illustrating results for two in Fig. 1a and b. We made amendments to the protocol of Wang *et al.* [5] based on concerns raised by Meng and Mao [6]. First, we added scores for *S. pani*; Wang *et al.* [5] did not score *S. pani* for the 509 morphological characters in their taxon-character matrix as little of the anatomy of this taxon beyond the ear ossicles was reported [2]. Second, we modified scores for the haramiyidans *Arboroharamiya* (regarding the shape of the incudal body) and *Qishou* (which was changed to unknown for all incudomallear characters), following Meng and Mao [6]. Wang *et al.* [5] suggested that *Qishou* (based on an image in [7]) had an incus and malleus much like that in *Vilevolodon*. Meng and Mao [6] include two computerized tomography (CT) slices of *Qishou* (Fig. 1k and l) that clearly show there is only one bone present. We acknowledge the error in interpretation by Wang *et al.* [5]. However, rather than an incus preserved on the malleus in *Qishou*, based on these new cross sections, we interpret this as the malleus with a facet for the incus, still resembling the condition in *Vilevolodon*. Nevertheless, we score *Qishou* as unknown here. Third, we eliminated the optimization of character 416, the alignment of the malleus and incus, because evaluation of this character requires knowledge of the plane of the ectotympanic, which is seldom preserved in fossils.

The first illustrated optimization is of character 417 from Wang *et al.* [5] (Fig. 1a), the shape of the incudal body, the part in contact with the malleus. We scored this as a trochlea in the outgroup *Morganucodon*, plate-like in most Mesozoic mammals, and globular in the extant therian *Philander* and *Arboroharamiya*, following Meng and Mao [6]. Wang *et al.* [5] (based on movies in [2]) suggested that the incus of the zhangheotheriid *Origolestes* had a thickened body and scored it as globular. We changed their score of *Origolestes* to plate-like; Meng and Mao [6] have provided a new CT slice of *Origolestes* (their Fig. 1i), which shows an incudal body more reminiscent of that in the monotreme *Tachyglossus* (their Fig. 1e) than in the marsupial *Didelphis* (their Fig. 1g). In our optimization (Fig. 1a), the plate-like incus is primitive for Mammalia with the globular state derived independently in *Philander* and *Arboroharamiya*. We found similar results for the amended optimizations for characters 415 and 418 (the geometry and orientation of the incudomallear joint, respectively), that is, the states associated with the condition in *Vilevolodon* and monotremes are primitive for Mammalia.

Character 419 concerns the extent of overlap between the malleus and incus, complete or partial, which is applicable only for taxa with the incudomallear articulation in the same plane as the mallear body (character 418). Our optimization (Fig. 1b) shows that the primitive condition for Mammalia is ambiguous, which differs from the results of Wang *et al.* [5] (with complete overlap as primitive) as well as Meng and Mao [6] (with partial overlap primitive in their Fig. 1o). This change from the conclusion of Wang *et al.* [5] is a result of the addition of scores for *S. pani* and removal of *Qishou*, which highlights how fluid such analyses are, given how few taxa are known for middle ear ossicles.

A finding of Wang *et al.* [5] that we emphasize here is the similarity of the incudomallear joint in multiple lineages of Mesozoic mammals and monotremes. We do not see major distinctions between the overlapping and partial overlapping joints and believe the transformation from one to the other did not require massive overhauling, contra Meng and Mao [6]. We illustrate this with the condition in the monotreme *Ornithorhynchus* (Fig. 1c and d). It has an overlapping joint (Fig. 1c), but the malleus has a low ridge that marks the edge of the incudomallear joint surface (arrows in Fig. 1d) and a joint surface that is partially concave (asterisk in Fig. 1d), both morphologies expressed in the partial overlapping joint. Transforming the condition of the platypus into the partial overlapping joint requires a posterior shift of the incus with respect to the malleus and a more pronounced ridge marking the incudomallear joint surface. These morphologies represent the first steps in the transformation of the load bearing trochlear joint between the quadrate (incus) and articular (malleus), as occurs in *Morganucodon* [8], for example. No matter what the direction of the transformation of the overlapping and partial overlapping joints may have been, it likely occurred more than once in mammal evolution, as the postdentary bones detached multiple times from the lower jaw [8].

## Supplementary Material

nwab132_Supplemental_File

## References

[1] Han G , Mao F-Y, Bi S-D et al. Nature 2017; 551: 451–6. 10.1038/nature24483 29132143

[2] Mao F-Y , Liu C-Y, Hill Chase M et al. Natl Sci Rev 2021; 8:nwaa188. 10.1093/nsr/nwaa188 PMC8288399 34691634

[3] Mao F , Hu Y, Li C et al. Science 2020; 367: 305–8. 10.1126/science.aay9220 31806694

[4] Luo Z-X , Meng Q-J, Grossnickle DM et al. Nature 2017; 548: 326–9. 10.1038/nature23483 28792934

[5] Wang J , Wible JR, Guo B et al. Nature 2021; 590: 279–83. 10.1038/s41586-020-03137-z 33505017

[6] Meng J , Mao FY. Natl Sci Rev 2021; 8: nwab131. 10.1093/nsr/nwab131 PMC8566185 34858616

[7] Meng J , Mao F, Han G et al. J Anat 2020; 236: 50–71. 10.1111/joa.13083 31498899 PMC6904648

[8] Luo Z-X. Annu Rev Ecol Evol Syst 2011; 42: 355–80. 10.1146/annurev-ecolsys-032511-142302

