# Supplementary material for: Response to ‘Monotreme middle ear is not primitive for Mammalia’
Source: Natl Sci Rev. 2021 Jul 23;8(10):nwab132. doi: 10.1093/nsr/nwab132 (PMC8566169; doi:10.1093/nsr/nwab132)
Supplement: nwab132_Supplemental_File [file nwab132_supplemental_file.docx]

Supplementary Information for

Response to “Monotreme middle ear is not primitive for Mammalia.”

John R. Wible^1,2^, Sarah L. Shelley^1,3^, and Shundong Bi^1,2,4^

1 Section of Mammals, Carnegie Museum of Natural History, Pittsburgh, Pennsylvania 15206, U.S.A.

2 Centre for Vertebrate Evolutionary Biology, Yunnan University, Kunming, China

3 School of Geosciences, University of Edinburgh, Edinburgh, EH9 3FE, United Kingdom

4 Department of Biology, Indiana University of Pennsylvania, Indiana, Pennsylvania 15705, U.S.A.

**Terminology issues.** It is generally accepted that three types of middle ears occur in the mammal lineage. (1) In the condition in extant mammals, the malleus, incus, and stapes are suspended in the middle ear and function exclusively in audition. This was called the definitive mammalian middle ear by Allin^9^ in 1975, which has been used by most subsequent authors. However, in 2019, Harper and Rougier^10^ proposed the alternative term detached middle ear. (2) In the ancestral condition, the postdentary bones (the articular [malleus], prearticular [gonial], angular [ectotympanic], and surangular) occupy a trough on the dentary, the quadrate (incus) has a broad contact on the skull base, and together these elements serve a dual function in jaw suspension and hearing, through their linkage with the stapes. Because this condition occurs in non-mammalian cynodonts, it has been referred to as the cynodont middle ear^9^, the mandibular middle ear of cynodonts^8^, or simply the mandibular middle ear^10, 11^, as preferred by Meng and Mao^6^. (3) After the term mandibular middle ear was coined, an intermediate condition was reported in some eutriconodontans and spalacotherioids wherein the postdentary bones did not have direct contact to the dentary but were indirectly connected through an ossified Meckel’s cartilage attached to the dentary^8, 12, 13^. This has been called the ‘transitional’ middle ear^12^, the partial mammalian middle ear^8^, the transitional mammalian middle ear^13^, and the partially detached middle ear^10^.

Meng and Mao^6^ want to use the terms definitive mammalian middle ear for condition #1 and mandibular middle ear for condition #2; they do not say which of the various terms for condition #3 that have been proposed they favor. As part of their rationale for retaining currently used terms for #1 and #2, Meng and Mao^6^ claim that because the current terms are long-standing and well defined, their continued usage fulfills the rule of priority. Our brief summary of the history of these terms above reveals that they are not particularly long-standing as various terms have been employed, even as recently as 2019. While we agree that new terms should only be added when the need arises, there is no rule of priority for anatomical terms. As Wang et al.^5^ are the second set of authors^9^ to propose new terms for these three conditions since 2019, it seems that the need is arising. Meng and Mao^6^ also suggest that the replacement terms offered by Wang et al.^5^ should be rejected because they are poorly defined. Wang et al.^5^ never suggested changing the definitions of the three conditions that all authors accept; they merely suggested changing the terms.

As noted previously^5, 10^, the term definitive mammalian middle ear for condition #1 has a taxon-based connotation that is problematic as a number of fossils that are phylogenetically mammals do not have the condition. Detached middle ear is a solution accepted by both Harper and Rougier^10^ and Wang et al.^6^ that we continue to support here.

Although mandibular middle ear for condition #2 is supported by both Meng and Mao^6^ and Harper and Rougier^10^, its usage has been made problematic with the discovery of the intermediate condition #3, which also has the middle ear attached to the mandible, in this case through Meckel’s cartilage. In our view, mandibular middle ear, therefore, does not sufficiently distinguish between conditions #2 and #3. Wang et al.^5^ proposed postdentary attached middle ear and Meckelian attached middle ear as a solution to distinguish conditions #2 and #3. Meng and Mao^6^ find the term postdentary attached middle ear intrinsically contradictory, because the postdentary bones are themselves the middle ear and not an attachment tool for the middle ear. We note that the postdentary bones are not the whole middle ear but only part, and in the case of condition #2 are the vehicle for the attachment of the whole middle ear. Another solution could be postdentary trough attached middle ear for condition #2 and Meckelian sulcus attached middle ear for condition #3 in order to be clearer, even if wordier. However, this does not avoid the issue that condition #2 has an attachment for the middle ear via the postdentary trough and Meckelian sulcus, whereas condition #3 has the Meckelian sulcus alone. The challenge is to coin descriptive terms that are unambiguous and terse. We continue to support the solution proposed by Wang et al.^5^, but whether other morphologists agree will only be known by future usages.

9 Allin, E. F. Evolution of the mammalian middle ear. *J Morphol* **147**, 403-437 (1975).

10 Harper, T. & Rougier, G. W. Petrosal morphology and cochlear function in Mesozoic stem therians. *PLoS ONE* **14**, e0209457 (2019).

11 Kielan-Jaworowska, Z., Cifelli, R. L. & Luo, Z-X. 2004. *Mammals from the Age of Dinosaurs: Origins,*

*Evolution, and Structure* (Cambridge Univ. Press, New York, 2004).

121 Luo, Z.-X., Chen, P., Li, G. & Chen, M. A new eutriconodont mammal and evolutionary development in early mammals. *Nature* **446**, 288–293 (2007).

13 Meng, J., Wang, Y. & Li, C. Transitional mammalian middle ear from a new Cretaceous Jehol eutriconodont. *Nature* **472**, 181–185 (2011).
